# Obesity-associated intestinal insulin resistance is ameliorated after bariatric surgery

**DOI:** 10.1007/s00125-015-3501-3

**Published:** 2015-01-29

**Authors:** Jaakko Mäkinen, Jarna C. Hannukainen, Anna Karmi, Heidi M. Immonen, Minna Soinio, Lassi Nelimarkka, Nina Savisto, Mika Helmiö, Jari Ovaska, Paulina Salminen, Patricia Iozzo, Pirjo Nuutila

**Affiliations:** 1 Turku PET Centre, University of Turku, PL 52, FIN-20520 Turku, Finland; 2 Department of Endocrinology, Turku University Hospital, Turku, Finland; 3 Department of Digestive Surgery and Urology, Turku University Hospital, Turku, Finland; 4 Institute of Clinical Physiology, National Research Council, Pisa, Italy

**Keywords:** Bariatric surgery, Insulin resistance, Intestine, Obesity, Positron emission tomography, Type 2 diabetes

## Abstract

**Aims/hypothesis:**

The intestine is the main site for glucose absorption and it has been suggested that it exhibits insulin resistance. Bariatric surgery has been shown to reverse insulin resistance and type 2 diabetes, but its effects on human intestinal metabolism are unknown. Our aim was to evaluate the effects of insulin on intestinal glucose metabolism before and after bariatric surgery.

**Methods:**

Twenty-one morbidly obese individuals undergoing bariatric surgery and ten age-matched healthy individuals were recruited and intestinal and skeletal muscle glucose uptake (GU) was measured using [^18^F]fluoro-2-deoxyglucose and positron emission tomography at fast and during hyperinsulinaemia. MRI was used as anatomical reference. Obese participants were studied again 6 months postoperatively.

**Results:**

In contrast to healthy individuals, insulin had no effect on intestinal GU in obese participants with or without diabetes, suggesting that intestinal insulin resistance is present early in morbid obesity. Postoperatively, jejunal GU increased in line with whole-body and muscle GU. Postoperative GU values in the intestine correlated with whole-body insulin sensitivity, indicating that the intestinal mucosa may reflect the overall glycaemic state and potentially mediate obesity-associated insulin resistance.

**Conclusions/interpretation:**

This study shows that insulin is a potent stimulator of GU in the healthy intestine and that intestinal insulin resistance is ameliorated after bariatric surgery. In our study, obese individuals had intestinal insulin resistance regardless of their glycaemic status. Persistent changes in intestinal glucose metabolism are likely to influence both local processes in the gut and systemic glucose homeostasis.

## Introduction

Understanding of the role of the intestine in glucose homeostasis is progressing with the increasing adoption of bariatric surgery in the management of morbid obesity. Bariatric surgery, originally intended to treat obesity, improves glycaemic control and/or reverses diabetes [1]. Roux-en-Y gastric bypass (RYGB), considered the standard technique, involves creating a small gastric pouch using a surgical stapler. This pouch is divided from the gastric remnant and anastomosed to the jejunum, allowing ingested food to bypass 95% of the stomach, the duodenum and the first portions of jejunum [1]. Alternatives to RYGB include sleeve gastrectomy (SG), in which a large portion of the stomach is removed along the greater curvature [2].

In a systematic review, Meijer and others report a type 2 diabetes mellitus reversal rate of 83% in patients undergoing the RYGB procedure [3]. Substantial weight loss occurs during the postoperative weeks and months, but the main metabolic changes occur rapidly and independently of weight loss [4]. Both insulin secretion and the improvement of insulin sensitivity are involved in the process. The increase in insulin sensitivity is a major early postoperative outcome of RYGB, whereas insulin secretion becomes affected at a later stage, possibly as an adaptation to the weight loss [5]. As with any intervention to treat diabetes, the residual beta cell function is a strong predictor of remission after bariatric surgery [6].

The intestine is a large organ and is responsible for glucose absorption. Among the mechanisms involved in the reversal of diabetes after surgery, the role of the entero–insular axis remains controversial [4, 7]. Two mechanisms are considered mainly responsible for the early improvement in glucose control: an increase in hepatic insulin sensitivity following energy restriction and an exaggerated secretion of glucagon-like peptide 1 (GLP-1) due to a more rapid exposure of nutrients to the distal jejunum [8].

Changes in the metabolic activity of the intestine after bariatric surgery remain unclear. Quantitative studies in humans are lacking, presumably because anatomical considerations limit non-invasive access to the organ. Positron emission tomography (PET) is a unique method of measuring metabolic rates in a specific tissue; the use of fluorine-18-labelled fluoro-deoxyglucose ([^18^F]FDG) in quantitative studies has been validated in several tissues and organs, such as the liver [9], skeletal muscle [10] and brain [11]. [^18^F]FDG is transported into cells, where it undergoes phosphorylation, but it cannot be further metabolised, enabling its accumulation rate in specific regions to be measured. We have recently validated the method to quantify intestinal glucose uptake (GU) against autoradiography and tissue samples, and demonstrated that within the intestine, most of the FDG accumulates in the mucosal layer [12]. Using this method, we observed that the intestinal mucosa is insulin resistant in human obesity. It is not known whether insulin resistance of the intestinal mucosa is reversed by bariatric surgery. The aim of this study was to evaluate the effects of insulin on intestinal mucosal GU before and after bariatric surgery in individuals with and without diabetes.

## Methods

### Participants

Morbidly obese individuals (*n* = 21, age 47.3 ± 9.4 years, BMI 43.2 ± 3.8 kg/m^2^), of whom eight had type 2 diabetes and five impaired fasting glucose (IFG) or impaired glucose tolerance (IGT) according to current guidelines [13], and age-matched healthy lean individuals (*n* = 10, age 47.3 ± 6.0 years, BMI 23.7 ± 1.8 kg/m^2^) were recruited (Table 1). This study was part of the larger multicentre study Sleevepass (ClinicalTrial.gov registration no. NCT00793143), which is a randomised multicentre study comparing two surgical techniques—RYGB and SG—in bariatric surgery [2].

**Table 1:** Anthropometric and metabolic characteristics of the study participants

Characteristic	Obese (*n* = 21)		Healthy (*n* = 10)
	Pre-operative	Postoperative	
Age (years)	47.3 ± 9.4		47.3 ± 6.0
Sex (M/F)	3/18		2/8
Type 2 diabetes	13 (2 IFG, 3 IGT)	6 (1 IFG, 1 IGT)	0
Weight (kg)	121 ± 11	93 ± 13^***^	69 ± 7^***^
BMI (kg/m^2^)	43 ± 4	33 ± 2^***^	24 ± 2^***^
HbA_1c_ (%) (mmol/mol)	5.9 ± 0.5 41 ± 5.5	5.6 ± 0.3^*^ 38 ± 3.3	5.7 ± 0.2 39 ± 2.2
FPG^a^ (mmol/l)	6.0 ± 0.8	5.4 ± 0.5^***^	5.3 ± 0.3^*^
*M* value^a^ (μmol kg^−1^ min^−1^)	12.8 ± 6.0	23.0 ± 8.5^***^	40.3 ± 9.5^***^

^a^Whole-body GU

FPG, fasting plasma glucose; M/F, male/female

*p < 0.05, **p < 0.01 and ***p < 0.005 vs pre-operative values

The obese participants recruited were consecutive patients referred to the Turku University Hospital for bariatric surgery. Inclusion criteria were BMI > 40 kg/m^2^ or > 35 kg/m^2^ with an additional risk factor, age 18–60 years and a history of non-successful carefully planned conservative treatments. Individuals using insulin treatment and/or with mental disorders, eating disorders, excessive use of alcohol or poor compliance were excluded, as were those with a body weight over 150 kg, because of restrictions of the imaging devices. For those with diabetes, the mean duration from onset was 3 years and the condition was already well treated, as the mean HbA_1c_ was 6.3 ± 0.3% (45.4 mmol/mol). All were using metformin. One participant was additionally using pioglitazone and sitagliptin, and one was using pioglitazone combined with metformin. The participants with IGT or IFG were not using any diabetes medication.

Healthy participants were recruited via an advertisement in local newspapers. They were not obese and had normal OGTT results.

Written informed consent was obtained after explaining the purpose and potential risks of the study to the individuals. The study protocol was approved by the Ethics Committee of the Hospital District of Southwest Finland and conducted according to the principles of the Declaration of Helsinki.

### Study design

Obese individuals were stratified to undergo RYGB or SG. The participants were imaged pre-operatively in two PET sessions. Imaging was complemented with MRI of the whole body performed on separate days in order to obtain an anatomical reference image. PET studies were performed either after an overnight fast or during a euglycaemic–hyperinsulinaemic clamp to study the stimulative effects of insulin on regional glucose use. The same studies were done in healthy individuals. Thereafter, obese participants followed a diet very low in energy (very low calorie diet; VLCD) for 1 month before the operation. The maximal daily energy intake was restricted to 3.35 MJ (800 kcal). Bariatric surgery was carried out and postoperative treatment administered according to the clinical protocol [2]. At 6 months postoperatively, all the imaging and laboratory assessments were repeated to study intestinal metabolism after surgery.

### PET studies

Obese individuals underwent four PET sessions: pre- and postoperative imaging studies were conducted both during a euglycaemic–hyperinsulinaemic clamp and in the fasting state. Participants were instructed to withhold from glucose-lowering medications 24–72 h prior to the metabolic studies. An overnight fast preceded all the imaging sessions. Participants were lying in supine position during the PET study. Two catheters were inserted: one in an antecubital vein for the infusions of insulin and glucose and for tracer injection, and the other in the opposite antecubital arterialised vein for blood sampling.

**Fig. 1:**
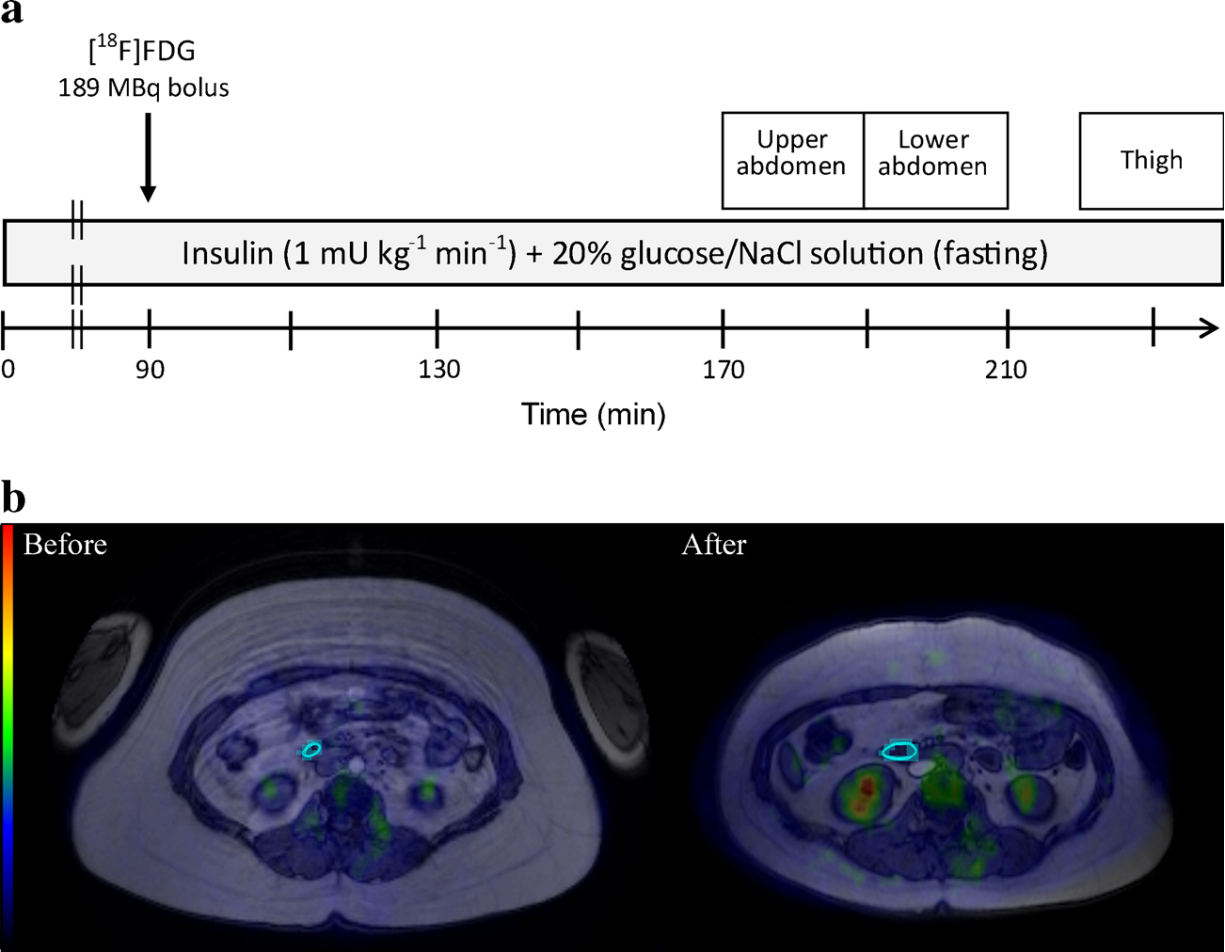
The imaging protocol and drawing of VOI. (**a**) In studies using hyperinsulinaemia, a euglycaemic–hyperinsulinaemic clamp was started for at least 180 min using intravenous insulin infusion. Euglycaemia was maintained using variable rates of 20% wt/vol. glucose infusion. In fasting studies, a 0.9% wt/vol. NaCl solution was used. At 90 min, [^18^F]FDG was injected and a dynamic scan of the upper abdominal region was started at 170 min followed by lower abdominal region at 190 min and femoral region at 220 min. (**b**) Axial slices of fused PET, MR images and VOI of the duodenum of an obese participant pre- and postoperatively. Luminal contents and highly active tissues were avoided in the analysis. VOI needed to be redrawn in the postoperative analysis because of altered intestinal anatomy and reduced overall fat mass

Levels of blood glucose, HbA_1c_, incretins and hormones were measured in all of the participants and again postoperatively in the obese participants. In studies using hyperinsulinaemia, a euglycaemic–hyperinsulinaemic clamp [14, 15] was started for 180 min using an intravenous insulin infusion (1 mU kg^−1^ min^−1^; Actrapid, Novo Nordisk, Copenhagen, Denmark). Euglycaemia was maintained using variable rates of a 20% wt/vol. glucose infusion, adjusted according to plasma glucose concentrations in arterialised blood, as measured every 10 min. At an average of 90 min, after the start of the study, [^18^F]FDG (mean 189 MBq, 147–218 MBq) was injected over 15 s and a dynamic scan of the upper abdominal region was started at 80 min (frames 5 × 180 s) followed by a similar scan of the lower abdominal region at 100 min and the femoral region at 130 min (3 × 300 s) (Fig. 1a). Arterial blood samples were drawn throughout the study after the [^18^F]FDG injection, and plasma radioactivity was assessed with an automatic gamma counter (Wizard 1480, Wallac, Turku, Finland).

### Image acquisition and processing

[^18^F]FDG was synthesised with an automatic apparatus as described by Hamacher et al [16]. PET images were acquired using the PET scanner GE Advance (General Electric Medical Systems, Milwaukee, WI, USA), which has a transaxial resolution of 3.8 mm (full width at half maximum [FWHM]) and slice width of 4.2 mm in the centre of the imaging field [17]. A transmission scan of 5 min was performed before the emission scan to correct for the tissue attenuation of gamma photons. All image data were corrected for dead time, decay and photon attenuation. MRI was performed using a Philips Intera 1.5 T scanner (Best, the Netherlands). The whole body of a participant was scanned with axial in-and-out-of-phase images with a repetition time of 120 ms, echo times of 2.3 ms and 4.63 ms, a slice thickness of 10 mm and a matrix 530 × 530 mm^2^.

### Analysis of PET images

Regional GU in the intestine was measured from the dynamic PET data using magnetic resonance (MR) images as anatomical reference, as recently described [12]. Localisation of the intestine was done on fused PET and MR images and the final localisation was always confirmed visually on the PET images. Both of the upper and lower abdominal imaging regions were used to reliably visualise and analyse parts of the intestine. Carimas 2.0.2 was used to manually draw three-dimensional hollow tubular volumes of interest (VOI) on sections of the descending duodenum and the jejunum. An example of this is shown in Fig. 1b. The tubular VOI were carefully shaped to contour the intestinal wall, avoiding intestinal contents and external metabolically active organs. Because of the reduced fat mass and altered gastrointestinal anatomy, VOI were redrawn in the postoperative images. Care was taken to draw the jejunal VOI on a section of the bowel distal to the Y-connection in the images of patients who had undergone RYGB surgery. In some cases, certain intestinal segments were not drawn and analysed because of uncertainty about the anatomy. Regions of interest were drawn on femoral muscles to assess skeletal muscle GU, as previously described [15].

A graphical method, the Gjedde–Patlak plot, was used to analyse [^18^F]FDG kinetics [18]. The use of this method to assess intestinal GU was validated in an animal model, in which PET data were compared with ex vivo tissue activity and autoradiography [12]. Regional time–activity curves (TACs) were obtained from the VOI. A regional fractional uptake rate (*K*_i_) was then calculated using each regional TAC and the plasma radioactivity curve. A VOI-specific GU rate was calculated by multiplying *K*_i_ by corresponding plasma glucose concentrations and dividing this product by a lumped constant (LC) of 1.30 in fasting studies and 1.15 in hyperinsulinaemic clamp studies [12]. An LC of 1.20 was used in the femoral skeletal muscle analysis [10]. The LC accounts for the differences in transport and phosphorylation between d-glucose and FDG and is used to convert the FDG uptake rate into the GU rate, expressed as μmol glucose min^−1^ kg tissue^−1^. Whole-body GU was calculated from the glucose infusion rate during PET in the hyperinsulinaemic–euglycaemic clamp, and expressed also in μmol min^−1^ kg^−1^.

### Biochemical analysis

Plasma glucose concentrations were measured in duplicate using the glucose oxidase method (Analox GM7 or GM9 Analox Instruments, London, UK). The level of HbA_1c_ was determined by HPLC (Variant II, Bio-Rad, Hercules, CA, USA). The Milliplex MAP Human Metabolic Hormone Panel was used to quantify the concentrations of active ghrelin, glucose-dependent insulinotropic polypeptide (GIP) and pancreatic peptide YY_3–36_ (PYY) and the Milliplex MAP Human Serum Adipokine Panel B was used for measuring leptin concentrations, following manufacturer’s instructions (Millipore Corporation, Billerica, CA, USA). The assay plates were analysed with Bio-Plex 200 System equipped with Bio-Plex Manager version 4.1 (Bio-Rad Laboratories, Solna, Sweden). Minimum detection limits for the active ghrelin, GIP, PYY and leptin were 2 pg/ml, 4 pg/ml, 7 pg/m and 85.4 pg/m, respectively.

Human GLP-1 and GLP-2 concentrations in lithium–heparin plasma samples were measured using a sandwich ELISA kit following the manufacturer’s instructions (Yanaihara Institute, Shizuoka, Japan). The optical density of the samples was determined using a Multiscan Ascent spectrophotometer (Thermo Labsystems, Helsinki, Finland). The minimum detection limits for the GLP-1 and GLP-2 assays were 0.206 and 0.412 ng/ml, respectively.

### Statistical analysis

Statistical analyses were performed using the SAS software for Windows version 9.2 (SAS Institute, Cary, NC, USA). Comparisons of non-paired data between two groups were made using a Student’s *t* test. A paired *t* test was used when comparing paired data. Pearson correlation coefficients were calculated where appropriate. All data are expressed as mean ± SD. Values of *p* < 0.05 were considered significant.

## Results

### Metabolic characteristics

Bariatric surgery resulted in a marked weight loss and improvement in glycaemic control (Table 1). Obese participants lost an average of 28 kg of weight in the 6 months following the operation and insulin sensitivity increased significantly, as shown by the whole-body GU rate (*M* value). The mean *M* value was increased by an average of 80% but remained low compared with the healthy participants (*p* < 0.005). Type 2 diabetes was in full remission—defined by normal plasma glucose in fasting conditions and during the OGTT [13]—in three of the eight participants with diabetes. Four participants still had diabetes and one had IFG. Of the five participants with IFG or IGT pre-operatively, four became euglycaemic and one developed diabetes. Metformin was still used by one participant. Diabetes medication was otherwise not used. Mean postoperative glycated haemoglobin was 5.9 ± 0.3% (41 mmol/mol) in the participants with a previous diagnosis of diabetes. There were no differences in participants’ glycaemic status following the two types of operation.

### The effect of insulin on intestinal GU in healthy participants

**Fig. 2:**
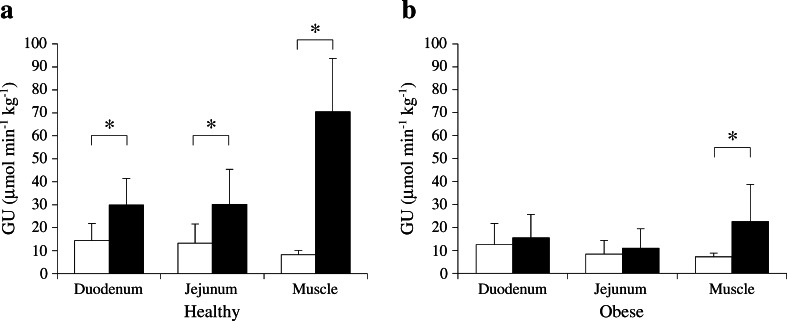
GU in the intestine and skeletal muscle. (**a**) Intestinal and muscular GU in the fasting state (white bars) and during euglycaemic–hyperinsulinaemia (black bars) in healthy participants. GU in both intestinal segments was significantly stimulated by insulin. (**b**) As in (**a**), but for the obese group (GU in fasting state, white bars; GU during hyperinsulinaemia, black bars). Insulin sensitivity was lacking in the intestine, whereas GU in femoral muscle was still augmented by insulin. *p < 0.05

Mean GU rates in the duodenum and jejunum were low after an overnight fast in the healthy participants (Fig. 2a) and in the same order of magnitude as in skeletal muscle. Insulin stimulation doubled intestinal GU from circulation (on average by 150% in the duodenum and 230% in the jejunum, *p* < 0.05 for both) (Fig. 2a). By comparison, skeletal muscle GU showed a 790% increase during hyperinsulinaemia (Fig. 2a).

### Intestinal GU in obese participants

**Fig. 3:**
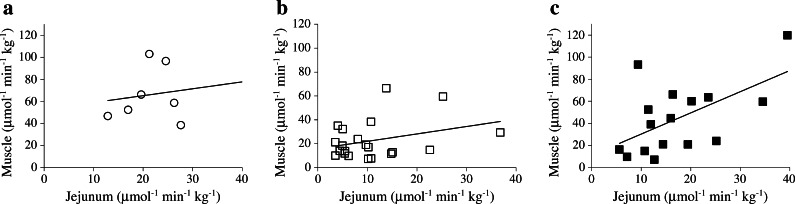
Correlations between jejunal and femoral muscle GU. (**a**) Jejunal GU did not correlate with the GU in femoral muscle in the healthy participants (*r* = 0.41, *p* = 0.28). (**b**) In obese participants, jejunal and muscular GU tended to correlate but failed to reach significance (*r* = 0.32, *p* = 0.15). (**c**) After bariatric surgery, jejunal and muscular GU correlated more strongly (*r* = 0.57, *p* < 0.05)

Before the operation, intestinal and skeletal muscle GU under fasting conditions did not differ from that in healthy participants (Fig. 2b). Obese patients were insulin resistant at the whole-body level (Table 1) and in line with this had blunted insulin-stimulated skeletal muscle GU compared with healthy individuals (Fig. 2b). The stimulatory effect of insulin on intestinal GU seen in healthy participants was lacking in obese patients (Fig. 2b). Jejunal GU tended to be associated with GU in femoral muscle in the obese, but this was not statistically significant (Fig. 3b). Correlations were also lacking in healthy participants (Fig. 3a) and the duodenum. Neither the intestinal nor the muscular GU differed between obese participants with or without diabetes.

### Effect of bariatric surgery on intestinal GU

**Fig. 4:**
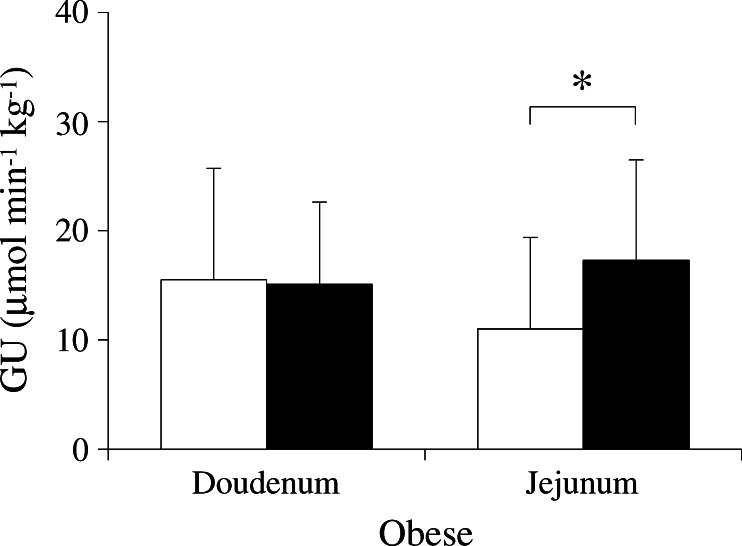
Effects of bariatric surgery on intestinal GU. Duodenal GU during hyperinsulinaemia remained at the same level postoperatively (black bars) as pre-operatively (white bars). Insulin-stimulated GU was increased by an average of 140% in the jejunum after bariatric surgery. *p < 0.05

Analysis of the studies performed 6 months postoperatively demonstrated no significant changes in duodenal GU in either the fasting or the hyperinsulinaemic state, compared with pre-operative values. The duodenum was still insulin resistant with a mean increase in GU during insulin stimulation of 39% (*p* = 0.08). Compared with the corresponding pre-operative values, jejunal GU was increased postoperatively on average by 130% during fasting (data not shown) and by 140% during hyperinsulinaemia (*p* = 0.049 and *p* = 0.005, respectively) (Fig. 4). Whole-body insulin sensitivity was significantly improved after the operation but still remained low compared with healthy individuals (Table 1). In line with this, muscle insulin-stimulated GU increased by 180% compared with the pre-operative value (*p* < 0.001). Postoperatively, jejunal GU was significantly associated with femoral muscle GU (Fig. 3c). Glucose tolerance was restored postoperatively in eight of the 13 participants with diabetes, IGT or IFG. This, however, did not affect the magnitude of increase observed in participants’ duodenal or jejunal GU. A one-way ANOVA with diabetes remission as the independent variable did not show a significant difference in the absolute change in GU in each of the intestinal segments between the glucose-tolerant and -intolerant participants who, pre-operatively, were diabetic and obese.

No significant differences in the operation-induced changes in intestinal GU were observed between the two surgical procedures. As assessed with a one-way ANOVA, the increase in insulin-stimulated GU in the jejunum tended to be higher in the participants who had undergone SG (13.0 ± 11.2 vs 3.9 ± 6.7 μmol min^−1^ kg^−1^, *p* = 0.055). Duodenal GU was unchanged with regard to the type of operation performed (*p* = 0.33). The use of metformin did not influence intestinal GU before or after surgery.

### Incretin and hormone levels

Fasting levels of GLP-1 and GLP-2 were similar between the obese and the healthy groups, and did not change after bariatric surgery (Table 2). Mean serum concentrations of GIP were 63% higher in obese participants than in healthy ones, but this difference fell short of statistical significance (*p* = 0.055). Postoperatively, the concentrations of GIP in obese patients were decreased and similar to the levels observed in healthy individuals (*p* = 0.33). No differences in PYY or ghrelin levels were present between the groups. In obese patients, the mean leptin concentrations were more than fourfold higher than in healthy participants at baseline and decreased postoperatively (Table 2).

**Table 2:** Summary of the measured hormones and incretins

Hormone/incretin	Obese (*n* = 21)		Healthy (*n* = 10)
	Pre-operative	Postoperative	
GLP-1 (ng/ml)	8.3 ± 2.6	8.0 ± 3.4	8.8 ± 2.1
GLP-2 (ng/ml)	11.0 ± 3.5	11.3 ± 3.8	11.8 ± 4.8
GIP (pg/ml))	22.3 ± 12.9	16.2 ± 7.0^†^	13.6 ± 6.1^‡^
PYY (ng/ml)	0.51 ± 0.95	0.38 ± 0.75	0.45 ± 1.0
Leptin (ng/ml)	56.0 ± 19.6	29.4 ± 16.3^***^	12.0 ± 7.9^***^
Ghrelin (pg/ml)	38.2 ± 33.3	34.8 ± 42.4	54.4 ± 51.9

***p < 0.0001, ^†^ *p* = 0.066 and ^‡^ *p* = 0.055 vs pre-operative values

Whole-body insulin sensitivity was negatively associated with the level of leptin (*r* = −0.74, *p* < 0.005) pre-operatively. No significant correlation was observed between pre-operative regional GU rates and incretins or hormones. Postoperatively, muscle GU in the fasting state correlated with the level of ghrelin (*r* = 0.89, *p* < 0.01). This was not observed during hyperinsulinaemia. No other significant correlations between postoperative GU and cytokine levels were found.

## Discussion

To our knowledge, these are the first results demonstrating quantitative changes in the glucose metabolism of the intestine in response to bariatric surgery in humans. Hyperinsulinaemia does not enhance GU from the circulation in morbidly obese insulin-resistant individuals, in contrast to its effects in healthy individuals. This indicates insulin resistance of intestinal enterocytes mucosa in the morbidly obese [12]. When obese individuals were re-studied 6 months after the operation, a small but significant improvement in jejunal, but not duodenal, glucose fluxes were observed.

Whether the difference in results is related to the type of operation performed and consequent anatomical and metabolic alterations or to the actual divergence in glucose metabolism between the intestinal segments is not immediately evident. Our data failed to show a significant divergence in intestinal GU between the two types of surgery, although jejunal GU tended to be higher in the patients who underwent SG.

There are differences in the physiology of the duodenum and jejunum. GLUT2 has been shown to be present in the apical membrane of jejunal enterocytes of diabetic individuals [19], and this has not been observed in duodenal biopsies [20]. Intestinal glucose absorption is largely regulated by changes in the location of GLUT2 in epithelial cells [21]. GLUT2 is rapidly translocated to the brush border membrane in response to dietary glucose. GLUT2 internalisation stimulated by insulin is the limiting factor for GU from the intestine [21]. Insulin-resistant humans have GLUT2 in a permanently apical location, which favours blood-to-lumen glucose flux during fasting hyperglycaemia and remarkably high GU from the lumen after a sugar-rich meal [19]. Our results support this hypothesis. The obese individuals would present with a permanently apical GLUT2 leading to a pathological blood-to-lumen glucose flux and decreased accumulation of glucose in the enterocytes. Based on this, bariatric surgery would lead to a healthier glucose metabolism in the jejunum by enabling the insulin-inducible internalisation of GLUT2 which would thus explain the higher postoperative jejunal GU. Duodenal GU responds to insulin in healthy individuals. As GLUT2 is absent in the apical membrane of duodenal enterocytes, their insulin-responsive glucose use is mediated by other pathways. Intestinal gluconeogenesis is most likely one of the major factors, as demonstrated by recent studies in mice and obese humans. This diet-induced intestinal gluconeogenesis has been proposed to mediate food intake and glucose homeostasis (e.g. endogenous glucose production via portal glucose sensing) [22, 23].

The main mechanisms suggested to account for early diabetes resolution after bariatric surgery include increased hepatic insulin sensitivity due to energy restriction and improved beta cell function. This is mainly a consequence of a more substantial secretion of GLP-1 from the distal small intestine [7]. Whether the increase in jejunal GU seen in our data directly promotes incretin secretion requires further study. In our results, patients were stratified according the change (or lack of change) in diagnostic category, from diabetes or IGT/IFG to normal glucose tolerance, but no significant difference in the respective changes in intestinal GU was found. The sample size of these subgroups was insufficient to detect differences based on categories. However, significant correlations were seen between intestinal and femoral muscle GU after surgery, which may indicate that intestinal insulin sensitivity helps promote the resolution of diabetes in an indirect fashion (i.e. via the improvement in whole-body GU). In our data, there was no correlation between intestinal and muscle GU in the healthy individuals, most probably because of the relatively low number of participants and the small variation between them.

PET is a highly sensitive method of quantitatively measuring tissue-specific metabolic rates. We have recently validated its use for the intestinal tract [12]. Some limitations still remain. The duodenum is fairly fixed in its location, but more distal intestinal segments shift with changes in abdominal pressure. This was addressed by selecting easily identifiable vertical segments of the intestine and confirming the location on PET and MRI while drawing VOI on the jejunum. Second, the transaxial resolution of PET in conjunction with the thinness of the intestinal wall might affect the results via two phenomena, spillover and partial volume. However, these effects proved modest in our previous validation study [12]. In the present study, the number of participants was relatively small because of the demanding protocol and limited scanning capacity. In addition, MR images from a separate day were used as an anatomical reference, causing some variability in anatomy between the imaging modalities. The obese individuals had a mean weight of 121 kg. As the most morbidly obese patients eligible for bariatric surgery were excluded, our results may have underestimated the metabolic effects of obesity. The participants were also already well treated with regard to diabetes, which explains the small difference in glucose levels between the obese and the healthy groups (Table 1). However, participants were found to have variability in regional and systemic insulin sensitivity.

In conclusion, this study shows that intestinal insulin resistance is ameliorated after bariatric surgery. In our data, obese individuals had intestinal insulin resistance regardless of their glycaemic status, suggesting that intestinal insulin resistance may occur before, and independently of, systemic insulin resistance. The results highlight that insulin is a potent stimulator of GU in the intestine, and that bariatric surgery induces significant metabolic changes in the gut. It is likely that persistent changes in intestinal glucose metabolism may have an influence on both local processes in the gut and systemic glucose homeostasis.

## Abbreviations

[^18^F]FDG: Fluorine-18-labelled fluoro-deoxyglucose
GIP: Glucose-dependent insulinotropic polypeptide
GLP-1: Glucagon-like peptide 1
GU: Glucose uptake
IFG: Impaired fasting glucose
IGT: Impaired glucose tolerance
*K*_i_: Fractional uptake rate
LC: Lumped constant
MR(I): Magnetic resonance (imaging)
PET: Positron emission tomography
PYY: Pancreatic peptide YY_3__–36_
RYGB: Roux-en-Y gastric bypass
SG: Sleeve gastrectomy
VOI: Volumes of interest
